# Syphilis testing adherence among women with livebirth deliveries: Indianapolis 2014-2016

**DOI:** 10.1186/s12884-021-04211-8

**Published:** 2021-10-30

**Authors:** Opeyemi C. Ojo, Janet N. Arno, Guoyu Tao, Chirag G. Patel, Brian E. Dixon

**Affiliations:** 1 Department of Epidemiology, Indiana University Richard M. Fairbanks School of Public Health, 1050 Wishard Blvd., RG 5, Indianapolis, IN 46202 USA; 2 Division of Infectious Disease, Department of Medicine, Indiana University School of Medicine, Indianapolis, IN USA; 3 Marion County Public Health Department, Health and Hospital Corporation, Indianapolis, IN USA; 4 Division of STD Prevention, Centers for Disease Control and Prevention, Atlanta, GA USA; 5 Center for Biomedical Informatics, Regenstrief Institute, Indianapolis, IN USA

**Keywords:** Congenital syphilis, Prenatal care, Prenatal screening, Women’s health

## Abstract

**Background:**

The number of congenital syphilis (CS) cases in the United States are increasing. Effective prevention of CS requires routine serologic testing and treatment of infected pregnant women. The Centers for Disease Control and Prevention (CDC) recommends testing all pregnant women at their first prenatal visit and subsequent testing at 28 weeks gestation and delivery for women at increased risk.

**Methods:**

We conducted a cross-sectional cohort study of syphilis testing among pregnant women with a livebirth delivery from January 2014 to December 2016 in Marion County, Indiana. We extracted and linked maternal and infant data from the vital records in a local health department to electronic health records available in a regional health information exchange. We examined syphilis testing rates and factors associated with non-testing among women with livebirth delivery. We further examined these rates and factors among women who reside in syphilis prevalent areas.

**Results:**

Among 21260 pregnancies that resulted in livebirths, syphilis testing in any trimester, including delivery, increased from 71.7% in 2014 to 86.6% in 2016. The number of maternal syphilis tests administered only at delivery decreased from 16.6% in 2014 to 4.04% in 2016. Among women living in areas with high syphilis rates, syphilis screening rates increased from 79.6% in 2014 to 94.2% in 2016.

**Conclusion:**

Improvement in prenatal syphilis screening is apparent and encouraging, yet roughly 1-in-10 women do not receive syphilis screening during pregnancy. Adherence to recommendations set out by CDC improved over time. Given increasing congenital syphilis cases, the need for timely diagnoses and prevention of transmission from mother to fetus remains a priority for public health.

**Supplementary Information:**

The online version contains supplementary material available at 10.1186/s12884-021-04211-8.

## Introduction

Congenital syphilis (CS) results from infection in infants in utero with the bacterium, *Treponema pallidum* [1]. Untreated, pregnant women with syphilis are at high risk of spontaneous fetal abortion, fetal death, and intrauterine growth retardation [1]. A fetus infected with syphilis can develop severe physical and neurological sequelae, including cardiac malformation, skeletal deformities, blindness, deafness, Hutchinson triad disorder, and hepatosplenomegaly [1]. However, the severity of these adverse health effects depends on the duration of the syphilis infection in the pregnant women and if and when treatment is initiated. As a result, early diagnosis, and treatment of syphilis in all pregnant women is imperative.

Although timely diagnosis and adequate treatment of syphilis infected pregnant women can prevent CS, the number of reported CS cases in the United States has more than tripled since 2013, rising from 362 in 2013 to 1306 in 2018, the highest number of cases in the past twenty years [2]. Similarly, CS has increased in Indiana, rising from zero cases between 2008 and 2013 to 30 cases between 2014 and 2018 [3]. Specifically in Marion County, the state’s most populous county, CS cases increased from zero in 2008 through 2015 to nine cases in 2016 through 2017 [4].

The increase seen in CS among infants is correlated with the increase in syphilis rate seen among women of reproductive age [2]. The Centers for Disease Control and Prevention (CDC) recommends screening all pregnant women for syphilis during their first prenatal visit as this is critical in CS prevention [5]. Women who engage in risky sexual behaviors, use illicit drugs, have STIs (sexually transmitted infections) or multiple sex partners during pregnancy or reside in communities with increased prevalence of syphilis infection are considered high risk and recommended to be screened again between 28 to 32 weeks and also at delivery [5]. These recommendations are supported by the American Congress of Obstetricians and Gynecologists (ACOG). Most states, including Indiana, require screening during the first trimester and subsequent screening in the third trimester if the women are considered high risk [6].

Despite established guidelines, pregnant women may still not be tested for syphilis. The CDC found that in 2018, 28.2% of mothers of infants with CS did not receive timely prenatal care. Of those women that did, 8.9% were not screened appropriately [7]. Late serologic diagnosis was not uncommon as 11.2% of women with CS infants had a negative test early in pregnancy and a positive one less than 30 days before or within 90 days after delivery. Alarmingly, 30.7% of women diagnosed with syphilis during pregnancy lacked timely treatment [7]. Studies have also shown that healthcare providers’ adherence to testing guidelines varies significantly from one infectious disease to the other, and that maternal factors such as insurance type and geographical location are factors that contribute to low adherence [8, 9]. Several studies have also noted significant differences in providers’ adherence level based on data source [8, 10–14]. The use of administrative data such as Medicaid data has shown providers’ adherence to prenatal syphilis testing to be 60% [13] while studies utilizing clinical records have found higher adherence rate over 80% [10, 11]. In a previous study we found that among confirmed stillbirth cases, just over 50% received any syphilis test [17]. In this study, using the same electronic medical record system, we examined providers’ adherence to CDC screening guidelines by examining the proportion of women with a livebirth delivery who received appropriate syphilis screening. Additionally, this study examined the factors associated with syphilis screening and non-screening among women with a livebirth delivery.

## Materials and methods

We conducted a cross-sectional cohort study on prenatal syphilis testing among women with a livebirth delivery in Marion County, Indiana, between January 1^st^, 2014 and December 31^st^, 2016. Pregnancies were identified using birth certificate data from the Marion County Public Health Department (MCPHD), which is responsible for collecting vital records on all births in the county. We linked birth certificate data with the mother’s demographic, clinical encounter, insurance, laboratory, census tract, and medical procedure data stored in the Indiana Network for Patient Care (INPC). The INPC, described previously [15], is a statewide network of electronic medical records that includes all major hospitals, laboratories, and many outpatient clinics in Indiana. The INPC is routinely used for health services and public health research [16], including prior studies on STIs [17–20]. Probabilistic matching techniques, described previously [21, 22], were used to match the mother’s social security number, last name, first name, date of birth, and gender from the birth certificate to her medical records.

For the purpose of this study, all live births with gestation age missing or greater than forty-two weeks were excluded (201 mothers) (See Supplemental Digital Content). Maternal age was restricted to the reproductive years of 15-44 years, 23 mothers were excluded based on this criteria. In addition, women without clinical encounter data, 94 mothers, in the INPC were excluded from the study, because these women lack pertinent data needed for analysis. Race categories were condensed to reflect the main racial strata in Indianapolis. During this investigation, many African Americans were erroneously coded as Native Hawaiians in one health system, although Native Hawaiians account for <.05% of the Indianapolis population [23]. Therefore we combined African Americans and Native Hawaiians into a single Black race category. Additionally, given low proportions in the underlying population, we combined Multiracial and Asian categories into a single Other race category.

The CDC recommends testing pregnant women for syphilis at the diagnosis of pregnancy and additional syphilis testing early in the third trimester and again at delivery for women who are at increased risk [24]. For this study, providers’ adherence to syphilis testing is determined by the timing of the test performed following testing recommendation from the CDC. Because individual-level risk factors were not available from the INPC, we classified women as high-risk using the syphilis prevalence associated with the zip code of the mother’s home address. During the study period, four zip codes consistently had the greatest number of syphilis cases, with prevalence ranging from 56 to 95 cases per 100000 compared to the overall rate in the Indianapolis Metropolitan statistical area (MSA) which ranged from 4.6 to 8.3 cases per 100000 during the study period. Mothers residing in these zip codes were designated high risk. A woman is classified as having syphilis (by serology) if a non-treponemal test and a treponemal test were both positive. In addition, a few cases were also classified as having syphilis by serology, where an IgG was positive with a positive (reactive) confirmatory test (± 7 days) such as an fluorescent treponemal antibody absorption (FTA-ABS) or T pallidum particle agglutination (TP-PA), even without a non-treponemal test.

The unit of analysis for this study is pregnancy; therefore, deliveries with multiple births (twins, triplets, etc.), were counted as one pregnancy. Additionally, two pregnancies in a year by the same mother was counted as two unique pregnancies. The conception date of each pregnancy was calculated by using the estimated gestational day and birthdate of the baby provided on the birth certificate. The trimester of the infant’s delivery was calculated using the estimated gestational days of the infant. We deduced the trimester of each lab test by taking the numbers of days from conception to the test date. A laboratory test was classified to be within first trimester if it was done at 13 weeks or earlier, second trimester if it was performed between 14 to 27 weeks, and third trimester as lab tests performed between 28 weeks and 42 weeks. Tests performed at delivery were defined as lab tests carried out seventy-two hours before or after the birth. To avoid overlapping of timelines, third trimester lab tests were classified as lab tests performed between 28 weeks and 72 hours before the infant’s birthdate.

Descriptive statistics were calculated to examine the characteristics of mothers with a live birth delivery. Testing rates were calculated by trimester, 30 days before delivery, and at delivery. Results were stratified by year, high risk status, testing status, and mothers with syphilis based on serology. Statistics included mean and standard deviation for continuous variables and frequency and proportion for categorical variables. To evaluate the association between non-testing of women during pregnancy while adjusting for potential confounders, we constructed a multiple logistic regression model with race, age, delivery status (preterm or full-term), risk status, and insurance. The statistical software SAS, version 9.4, (SAS Institute Cary, NC) was used for all analyses, and a P-value < 0.05 was used for the level of significance. The study received approval by the Institutional Review Board at Indiana University (Study No. 1311659626 and 1611016230).

## Results

### Study population

We identified a total of 21260 pregnancies by 19574 unique mothers from 2014 to 2016. Demographics of the study populations are given in Table 1. Because we were most interested in testing during pregnancy, demographics are given as characteristics of pregnancies rather than characteristics of mothers themselves. Of the pregnancies, 52.0% were among White women, 31.7% were among Black women, and 9.3% were among women of mixed or other race. Ethnically, 5.9% were Hispanic, 57.6% non-Hispanic with the rest unknown or other. Most of the women were 20 to 34 years of age, with mean age being 27 years. There were 6,309, 7,946, and 7,006 pregnancies in 2014, 2015, and 2016, respectively.

**Table 1:** Prenatal syphilis testing among pregnancies with live birth deliveries^a^ by race, ethnicity, age, insurance and risk, Central Indiana, 2014–2016 (*N*=21,260)

		Total Pregnancies		
Characteristics		Total N (% of Pregnancies)	Syphilis Testing (% Tested/Total)	Syphilis “Cases” ^b^ (% Positive/Total)
**Race**				
White		11,052 (52.0%)	8,325 (75.33%)	13 (0.12%)
Black		6,729 (31.7%)	6,088 (90.47%)	20 (0.30%)
Other		2,028 (9.3%)	1,660 (81.85%)	6 (0.30%)
Unknown not documented		1,451 (6.8%)	1,294 (89.18%)	2 (0.14%)
**Ethnicity**				
Hispanic		1,260 (5.9%)	1,162 (92.22%)	2 (0.16%)
Non-Hispanic		12,244 (57.6%)	10,857 (88.67%)	29 (0.24%)
Unknown, not reported, other		7,756 (36.5%)	5,348 (68.95%)	14 (0.18%)
**Age group**				
15-19		1,755 (8.3%)	1,514 (86.27%)	1 (0.06%)
20-24		6,259 (28.4%)	5,285 (84.44%)	5 (0.08%)
25-29		6,292 (29.6%)	5,078 (80.71%)	9 (0.14%)
30-34		4,588 (21.6%)	3,621 (78.92%)	12 (0.26%)
35-39		1,965 (9.2%)	1,556 (79.19%)	10 (0.51%)
40-44		401 (2.0%)	313 (78.05%)	4 (1.00%)
**Delivery status**	Preterm	2,093 (9.8%)	1,789 (85.45%)	9 (0.43%)
	Full-term	19,167 (90.2)	15,578 (81.28%)	32 (0.17%)
**Insurance**	Government	12,625 (59.4%)	11,040 (87.45%)	30 (0.24%)
	Commercial	6,334 (29.8%)	4,506 (71.14%)	4 (0.06%)
	Self-pay	1,197 (5.6%)	1,068 (89.22%)	5 (0.42%)
	Other	1,104 (5.2%)	753 (68.21%)	2 (0.18%)

^a^Mothers were counted twice or more if they had multiple pregnancies during the time period

^b^Syphilis “Cases” are those with positive serologic screening and confirmatory tests regardless of history, clinical signs or treatment and so do not reflect women with active syphilis during pregnancy

Maternal prenatal syphilis testing was performed for 17,367 pregnancies (81.7%). This includes testing done any time during the pregnancy or at the time of delivery. Testing was done in 75.3% of pregnancies among White women compared to 90.4% of pregnancies among Black women and 81.9% of pregnancies of women of Mixed or Other race and 89.2% of pregnancies of women whose race was unknown. Testing was performed in 92.2% of pregnancies among Hispanic women, 88.7 and 68.9% of pregnancies among women who were non-Hispanic and whose ethnicity were unknown respectively. High risk was identified in 3,705 (17.4%) of pregnancies. Testing was performed in 89.4% of pregnant women identified as high risk compared to 80% of women not identified as high risk.

### Timing of maternal prenatal testing

Maternal testing was performed in 60.1% of pregnancies during the first or second trimesters (Table 2). The proportion of pregnant women tested in the first or second trimesters increased from 37.8% in 2014 to 75.5% in 2016. Testing occurred only at the time of delivery in 9.1% of pregnancies and this proportion decreased from 16.6% in 2014 to 4.0% in 2016. Among all pregnancies, 70.7% had syphilis testing performed at least thirty days before delivery, increasing from 51.8% in 2014 to 81.3% in 2016 (Table 2). Overall, the proportion of pregnancies that had syphilis testing in any trimester plus delivery increased from 71.7% in 2014, 85.3% in 2015 to 86.6% in 2016.

**Table 2:** Timing of maternal syphilis testing and serologic diagnoses in total and high-risk populations

Deliveries	Maternal testing N (%)				2014 N (%)				2015 N (%)				2016 N (%)			
Time of syphilis test	Total^a^	Positive^b^	High-Risk^f^ Total^c^	High-Risk Cases^d^	Total^a^	Positive^b^	High-Risk Total^c^	High-Risk Cases^d^	Total^a^	Positive^b^	High-Risk Total^c^	High-Risk Cases^d^	Total^a^	Positive^b^	High-Risk Total^c^	High-Risk Cases^d^
First trimester	8,308 (39.1%)	20 (48.8%)	1,569 (42.4%)	5 (55.6%)	1,122 (17.8%)	7 (43.8%)	235 (21.0%)	3 (42.9%)	3,446 (43.4%)	7 (50%)	684 (48.4%)	2 (100%)	3,740 (53.4%)	6 (54.5%)	650 (55.5%)	0
First trimester only^e^ (minus second and third)	798 (3.8%)	0	147 (4.0%)	0	152 (2.4%)	0	28 (2.5%)	0	333 (4.2%)	0	61 (4.3%)	0	313 (4.5%)	0	58 (5.0%)	0
First trimester or second trimester	12,782 (60.1%)	32 (78.1%)	2,498 (67.4%)	5 (55.5%)	2,385 (37.8%)	9 (56.3%)	481 (42.9%)	3 (42.9%)	5,109 (64.3%)	14 (100%)	1,043 (73.8%)	2 (100%)	5,288 (75.5%)	9 (81.8%)	974 (83.2%)	0
Second trimester	7,028 (33.1%)	25 (61.0%)	1,442 38.9%)	2 (22.2%)	1,671 (26.5%)	7 (43.8%)	333 (29.7%)	1 (14.3%)	2,744 (34.5%)	11 (78.6%)	578 (40.9%)	1 (50%)	2,613 (37.3%)	7 (63.6%)	531 (45.3%)	0
Second trimester only^e^ (minus first and third)	2,099 (9.9%)	3 (7.3%)	430 (11.6%)	0	748 (11.9%)	1 (6.3%)	147 (13.1%)	0	765 (9.6%)	1 (7.1%)	165 (11.7%)	0	586 (6.9%)	1 (9.1%)	118 (10.1%)	0
Third trimester	10,193 (47.9%)	29 (70.7%)	1,926 (52.0%)	6 (66.7%)	2,192 (34.7%)	10 (62.5%)	436 (38.9%)	5 (71.4%)	4,083 (51.4%)	11 (78.6%)	793 (56.1%)	1 (50%)	3,918 (55.9%)	8 (72.7%)	697 (58.7%)	0
Third trimester only^e^ (Minus 1^st^ and 2^nd^)	2,647 (12.5%)	6 (14.6%)	471 (12.7%)	2 (22.2%)	1085 (17.2%)	4 (25.0%)	209 (18.7%)	2 (28.6%)	1067 (13.4%)	0	173 (12.2%)	0	495 (7.1%)	2 (18.2%)	89 (7.6%)	0
≥30 days before delivery	15,027 (70.7%)	36 (87.8%)	2,874 (77.6%)	7 (77.8%)	3,270 (51.8%)	13 (81.3%)	645 (57.6%)	5 71.4%)	6,060 (76.3%)	14 (100%)	1,185 (83.8%)	2 (100%)	5,697 (81.3%)	9 (81.8%)	1,044 (89.2%)	0
Any trimester plus delivery	17,367 (81.7%)	41 (100%)	3,302 (89.1%)	9 (100%)	4,520 (71.7%)	16 (100%)	891 (79.6%)	7 (100%)	6,781 (85.3%)	14 (100%)	1,308 (92.5%)	2 (100%)	6,068 (86.6%)	11 (100%)	1,103 (94.2%)	0
At delivery only	1,938 (9.1%)	3 (7.3%)	333 (9.0%)	2 (22.2%)	1,050 (16.6%)	3 (18.8%)	201 (18.0%)	2 (28.6%)	605 (7.6%)	0	92 (6.5%)	0	283 (4.0%)	0	40 (3.4%)	0
Total	**21,260**	**41**	**3,705**	**9**	**6,309**	**16**	**1,120**	**7**	**7,945**	**14**	**1,414**	**2**	**7,006**	**11**	**1,171**	**0**

^a^ Total, indicates the overall study population

^b^ Positive, indicates mothers in the overall study population classified as syphilis cases. Note that it was not possible to determine the difference between current and prior infection among the syphilis cases

^c^ High risk total, indicates the overall population that resided in areas with high prevalence of syphilis

^d^ High risk cases, indicates the mothers in the overall high-risk population classified as syphilis cases

^e^ Mothers were only tested in the trimester indicated and not in any other trimester

^f^ High Risk was defined based on women residing in zip codes with high prevalence of syphilis

Forty-one pregnancies were classified as having syphilis infection: 16 (39.0%) of these were in 2014, 14 (34.2%) in 2015, and 11 (26.8%) in 2016. Testing among these cases increased from 48.8% during the first trimester to 70.7% at the third trimester, however, 7.6, 7.3 and 14.6% were solely tested at first, second, and third trimesters respectively without indication of testing in any other trimester. Overall, 36 (87.8%) pregnant women were diagnosed at least 30 days before delivery and 3 (7.3%) were diagnosed at delivery.

**Fig. 1:**
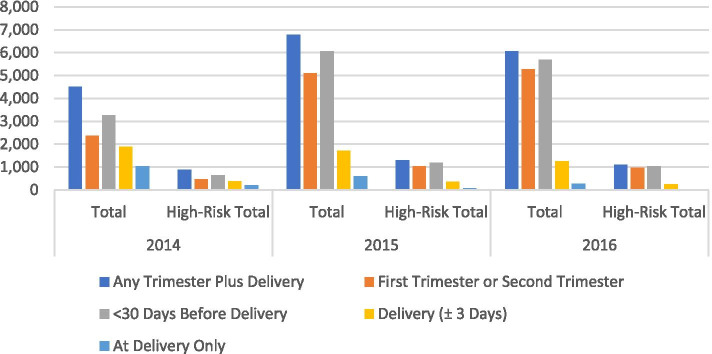
Count of maternal prenatal syphilis testing 30 days before delivery and any trimester plus delivery in the total population and high-risk sub-population from 2014-2016

We next examined whether testing occurred differently in pregnancies among women who were classified as high risk on the basis of residence in a high syphilis prevalence zip code compared to those who did not reside in high prevalence zip codes (Table 2). Among 3705 pregnancies of women from high syphilis prevalent zip codes, 2498 (67%) had testing in the first or second trimesters. Testing in the first or second trimesters increased among pregnancies of women from high risk zip codes from 42.9% in 2014, 73.9% in 2015 to 81.8% in 2016. Overall, 2874 (77.6%) pregnancies had prenatal syphilis testing performed at least thirty days before delivery (Fig. 1). The proportion of high-risk pregnancies that had prenatal syphilis testing anytime during pregnancy plus delivery increased from 79.6% in 2014, 92.5% in 2015 to 94.2% in 2016. The proportion of these pregnancies tested increased from 42.4% during the first trimester to 52% at the third trimester, however, 4, 11.6 and 12.7% were solely tested at first, second, and third trimesters respectively without indication of testing in any other trimester.

Of the 41 women classified as having syphilis, only nine resided in high prevalence zip codes of which seven were tested at least 30 days before delivery. Two were tested only at the time of delivery. Seven of these cases were in 2014, two in 2015, and none were reported in 2016. The testing that occurred only at delivery was only observed in 2014; subsequent years did not have syphilis testing occurring only at the time of delivery.

### Associated risk factors

We examined some of the potential factors that could contribute to lack of prenatal syphilis testing, including age, race, ethnicity, insurance, risk based on high syphilis prevalence zip codes, and delivery status. Bivariate analysis showed that all factors were significantly associated with prenatal syphilis testing (*P*<0.0001). When modeled using multiple logistic regression, which controls for other covariates, all factors except age (*P*=0.8832) remained significant predictors of syphilis testing during pregnancy (Table 3). Mothers that live in high syphilis morbidity areas had an adjusted odds ratio of 1.384 (*P*<0.0001), indicating that these women are more likely to have prenatal syphilis testing when compared to those that reside in non-high-risk zip codes. Mothers who were Black, Other, and whose race were unknown, when compared to White mothers had an adjusted odds ratio of 2.162 (*P*<0.0001), 1.197 (*P*=0.0057), and 2.021 (*P*<0.0001) respectively. Similarly, mothers with pre-term deliveries were more likely to have prenatal syphilis testing than mothers with full-term deliveries (adjusted odds ratio of 1.195 (*P*=0.0089)). Individuals with government insurance such as Medicaid and Medicare or self-pay for their healthcare utilization were more likely to have prenatal syphilis testing compared to those with commercial insurance with the adjusted odds ratio of 1.848 (*P*<0.0001) and 1.722 (*P*<0.0001) respectively.

**Table 3:** Results of the multiple logistic regression analyses by potential contributing factors

	Adjusted odds ratio (***P***-value)
**Characteristics**	
**Race**	
White	Ref.^a^
Black	2.162 (<0.0001)
Other	1.197 (<0.0057)
Unknown not documented	2.021 (<0.0001)
**Delivery status**	
Full-term	Ref.
Preterm	1.195 (0.0089)
**Insurance**	
Commercial	Ref.
Government	1.848 (<0.0001)
Self-pay	1.722 (<0.0001)
Other	0.793 (<0.0017)
**High risk status**	
No	Ref.
Yes	1.384 (<0.0001)

^a^ Ref. indicates the reference group used in the regression model

## Discussion

Prenatal syphilis testing is an essential preventative measure to halt the transmission of maternal syphilis infection to the fetus, which can lead to congenital syphilis in infants and stillbirths [1]. In this study, providers tested 82% of pregnancies that resulted in live births from 2014 to 2016. Providers’ adherence increased over time from 71.1% in 2014 to 86.6% in 2016.

Although women who were classified as high risk were screened more frequently, adherence to screening at 28 weeks and delivery was poor. In agreement with previous studies, we found that those with government insurance such as Medicaid and Medicare were tested more frequently than women with commercial insurance [25].

Providers’ adherence in this study was lower than previous studies [8–10] including one from Indianapolis [13] (95-98.2%). These studies were published over 15 years ago. Although there were differences in study populations, it is also possible that providers decreased testing practice after CDC’s efforts to eliminate syphilis beginning in 1999 [26].

We found that 19% of women were not tested for syphilis at any time during pregnancy. These are missed opportunities to detect and treat syphilis infections. A Louisiana study examining opportunities to prevent CS found that one-third of CS cases could have been prevented if prenatal syphilis testing was done [27]. Another study found that over one-third of women who had a stillbirth were not screened for syphilis [17, 28]. Diagnoses must be followed by timely treatment. However, Kidd et al. (2018) found that most infants with congenital syphilis were born to mothers who tested positive and were diagnosed with syphilis but not treated [29]. Our study was unable to assess treatment because treatment data is incomplete in the INPC.

In addition, our study also indicates that 41 women had syphilis infection based on serology from 2014 to 2016. In Marion County, during this time, there were 365 cases of early syphilis, of which 24 were women, and 189 late latent syphilis cases, of which 66 were women [4]. In the state, a total of 23 congenital syphilis cases were reported during the study period [3, 30]. Specifically, in Marion County, a total of 5 CS cases were observed between 2014 and 2016, with these cases occurring in 2016. Although we found five pregnancies with serologic diagnosis of syphilis less than thirty days before delivery, we cannot confirm that these are the same CS cases reported because our data were de-identified.

Contrary to Schrag et al. (2003), we found that providers were more likely to test women at higher risk, as defined by zip code of residence, than the overall population [8]. The increase in testing observed within the high-risk population is consistent with the CDC prenatal testing guidance [24]. However, despite the recommendation from the CDC and ACOG, only 27% of high-risk women were tested at delivery, and 9% were tested only at delivery. Alarmingly, our study found that over 7.3% of the women that tested positive for syphilis during the study period were tested only at delivery. These data indicate that providers do not fully adhere to testing recommendations when attending to women that reside in areas with high rates of syphilis. This low compliance with the CDC and ACOG guidelines on high-risk prenatal syphilis testing suggests that more providers education is needed.

Providers were more likely to test women with government-issued insurance, had a preterm delivery, resided in high-risk zip codes, and self-identified as Black. Similarly, Sheikh et al. (2008), found socioeconomic factors such as maternal residence, lack of healthcare insurance, and incarceration to be associated with lack of prenatal syphilis testing [9]. We found that those with government insurance such as Medicaid and Medicare were tested more frequently than women with commercial insurance, which is on par with the 85% testing rate among pregnant women found in a study using MarketScan data [25]. Government-funded health insurance afford women the opportunity to seek and receive care during pregnancy. Women who delivered preterm were also tested more often, possibly because of more frequent follow-up appointments associated with preterm risk. We were, however, unable to assess the reasons for preterm birth.

There were several limitations in this study. First, syphilis testing may have taken place in a lab not covered by the INPC, in which case a woman would appear not to be tested [13]. Additionally, results are dependent on institutions entering data correctly. Another limitation of this study is that we could not ascertain the stages of the syphilis diagnosis. Thus, it is possible that cases were from a previous infection that occurred before pregnancy that might not represent active syphilis. Additionally, the definition of high risk for this study did not encompass the entire definition of high risk as outlined by the CDC which includes individuals with risky sexual behavior, illicit drug use, STIs, or multiple sex partners during pregnancy [5]. Thus, it is possible for some women that were not classified as high risk based on the definition used in this study (high morbidity zip codes) to, in fact, be high risk. Lastly, assigned Logical Observation Identifiers Names and Codes (LOINC) coding to some laboratory tests was unclear as to which syphilis test was performed. Although all LOINCs with positive results were examined manually, it is still possible that misclassification could have occurred.

## Conclusions

Prenatal syphilis testing is crucial to reduce infant mortality and morbidity. In comparison to earlier studies, providers’ adherence to prenatal syphilis screening is relatively low. However, our study found an overall increase in testing over time which aligned with CDC’s call to action for providers to increase testing due to increase in congenital cases [31]. It is imperative to increase screening among high-risk populations, particularly at 28 weeks and at delivery. Future studies to examine treatment data and explore the proportion of pregnant women with syphilis that meet CDC’s high-risk definition is warranted.

## Supplementary Information

**Additional file 1.** Supplemental Digital Content

## Data Availability

The data that support the findings of this study are available from Regenstrief Institute, but restrictions apply to the availability of these data, which were used under license for the current study, and so are not publicly available. Data are however available from the authors upon reasonable request and with permission of Regenstrief Institute.
