# Supplementary figures and images for: Syphilis testing adherence among women with livebirth deliveries: Indianapolis 2014-2016
Source: BMC Pregnancy Childbirth. 2021 Oct 30;21:739. doi: 10.1186/s12884-021-04211-8 (PMC8557034; doi:10.1186/s12884-021-04211-8)

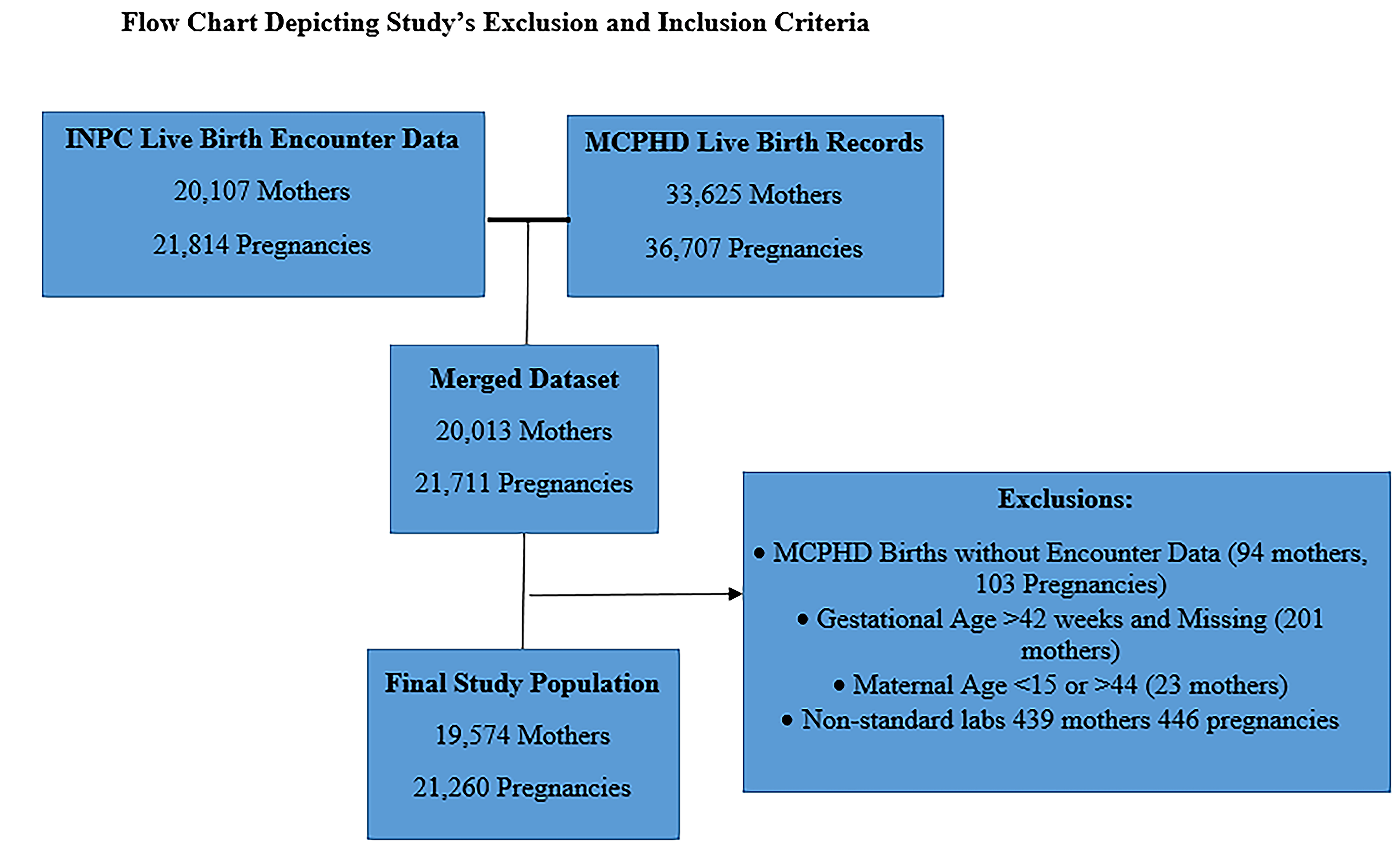

Supplement: Supplementary file 1 — Additional file 1. Supplemental Digital Content [file 12884_2021_4211_MOESM1_ESM.tif]
